# Effects of SGLT2 inhibitors on eGFR in type 2 diabetic patients—the role of antidiabetic and antihypertensive medications

**DOI:** 10.1038/s41440-020-00590-1

**Published:** 2020-12-14

**Authors:** Koichi Kitamura, Koichi Hayashi, Shinsuke Ito, Yuiko Hoshina, Masahiro Sakai, Kaede Yoshino, Keita Endo, Shigeki Fujitani, Toshihiko Suzuki

**Affiliations:** 1 Tokyo Bay Urayasu Ichikawa Medical Center, Chiba, Japan; 2 Department of Internal Medicine, Tokyo Dental College, Chiyoda City, Japan; 3 Department of Emergency and Critical Care Medicine, St Marianna University School of Medicine, Kawasaki, Japan

**Keywords:** Ca^2+^ channel blockers, eGFR, metformin, renin-angiotensin system inhibitors, renal microvessels, SGLT2 inhibitors

## Abstract

Recent randomized trials demonstrating the beneficial effects of sodium-glucose cotransporter 2 inhibitors (SGLT2is) in type 2 diabetes suggest that early reductions in eGFR upon initiation of SGLT2i therapy are associated with improved renal outcomes. Multiple concomitant medications, including antidiabetic and antihypertensive agents, are commonly used, however, which may modify the renal hemodynamic action of SGLT2is. Here we found that background treatment with metformin diminished the SGLT2i-induced reductions in eGFR after 3 months of SGLT2i therapy in patients with type 2 diabetes and hypertension (−2.29 ± 0.90 vs −5.85 ± 1.27 mL/min/1.73 m^2^ for metformin users (*n* = 126) and nonusers (*n* = 97), respectively). Other antidiabetic agents (DPP4 inhibitors, sulfonylureas and insulin) had no effect on the eGFR response to SGLT2is. Antihypertensive drugs, including calcium channel blockers (CCBs) and β blockers, did not affect the SGLT2i-induced changes in eGFR, whereas renin-angiotensin system inhibitors (RASis) tended to enhance this response (*p* = 0.059). Next, we evaluated the interaction between metformin and RASis in the eGFR responses to SGLT2is. Under no background treatment with RASis, metformin abrogated the eGFR response to SGLT2is, but this response was preserved when RASis had been given along with metformin (decreases of 0.75 ± 1.28 vs. 4.60 ± 1.15 mL/min/1.73 m^2^ in eGFR, *p* = 0.028). No interaction between metformin and insulin or between metformin and DPP4 inhibitors was observed. In conclusion, metformin blunts the SGLT2i-induced decrease in eGFR, but coadministration of RASis ameliorates this response. Furthermore, the inability of CCBs to modify the SGLT2i-induced reduction in eGFR suggests that the SGLT2i-induced renal microvascular action is mediated predominantly by postglomerular vasodilation rather than preglomerular vasoconstriction.

## Introduction

Diabetes mellitus (DM) not only causes detrimental effects on cardiovascular integrity but also aggravates renal function, which should lead to end-stage kidney disease requiring renal replacement therapy. Recent advances in pharmaceutical sciences facilitate the development of new classes of antidiabetic drugs, including dipeptidyl peptidase 4 (DPP4) inhibitors, glucagon-like peptide-1 (GLP-1) receptor agonists and sodium-glucose cotransporter 2 (SGLT2) inhibitors. Whereas a variety of conventional antidiabetic drugs have been shown to retard the progression of nephropathy through glycemic control [1], there are a limited number of observations demonstrating the direct salutary action of antidiabetic drugs on the progression of nephropathy [2]. Recently, convincing evidence has accumulated that SGLT2 inhibitors offer direct renal protective action, including alleviation of albuminuria and prevention of worsening nephropathy [3–6]. Although it is generally accepted that SGLT2 inhibitors reduce blood pressure (BP) and body weight (BW) along with blood glucose, the alterations in these factors cannot fully explain the renal benefits observed in patients with DM nephropathy, and the mechanisms whereby SGLT2 inhibitors improve renal outcomes are not well understood. Alternatively, it has been established that SGLT2 inhibitors cause an acute decrease in estimated glomerular filtration rate (eGFR), which remains stable thereafter and is reversible upon cessation of the therapy [3, 4, 6]. These findings could lend support to the conjecture that the beneficial action of SGLT2 inhibitors is attributed in part to the mitigation of glomerular hypertension, the mechanism of which is allegedly mediated by enhanced tubuloglomerular feedback (TGF) resulting from increased delivery of sodium chloride to the macula densa and causing afferent arteriolar constriction [7].

DM triggers a chain of diseases, including hypertension and dyslipidemia, and requires multiple drug therapies. Hypertension, in particular, constitutes an important therapeutic target because it causes renal damage and accelerates the progression of DM nephropathy. It has been established that renin-angiotensin system (RAS) inhibitors are the treatment of choice because of their unique pharmacological characteristics, i.e., the ability to reduce not only systemic BP but also glomerular pressure. Furthermore, calcium channel blockers (CCBs) are administered as an add-on therapy for strict BP control. Notably, in the EMPA-REG OUTCOME trial, approximately 80% of the subjects had been given RAS inhibitors, and 27.8–45.8% received CCBs as background medications to SGLT2 inhibitors [3]. These findings prompted us to speculate that the renal hemodynamic effects of SGLT2 inhibitors are modified by background medications such as RAS inhibitors and CCBs, both of which possess vasodilator action on renal microvessels. Finally, the treatment of type 2 DM usually requires multiple antidiabetic drugs, among which metformin is recognized as a first-line drug. Intriguingly, recent post hoc analyses of the EMPA-REG OUTCOME [8] and the CANVAS [9] trials have demonstrated that there are greater reductions in risk for worsening nephropathy and heart failure in patients not using metformin than in those using metformin at baseline. Nevertheless, the acute effects of SGLT2 inhibitors on renal function under metformin therapy have not been scrutinized hitherto.

We therefore conducted a retrospective survey evaluating the effect of SGLT2 inhibitors on eGFR over the 12-month study period and examined whether this effect was modified by other antidiabetic and/or antihypertensive drugs in patients with type 2 DM. The present study demonstrates close interplay among SGLT2 inhibitors, metformin and RAS inhibitors, all of which are commonly used together for glycemic and BP control. SGLT2 inhibitor-induced decreases in eGFR, allegedly associated with alleviation of glomerular hypertension, were diminished by metformin, but this response can be restored by further addition of RAS inhibitors. These observations thus merit comprehensive consideration with regard to the renal hemodynamic action of SGLT2 inhibitors and their background medications when SGLT2 inhibitors are used in patients with DM and hypertension.

## Methods

This study is a retrospective analysis evaluating the role of antihypertensive and antidiabetic agents on the renal hemodynamic effects of SGLT2 inhibitors in type 2 DM. The study was approved by the Ethics Committee of Tokyo Bay Urayasu-Ichikawa Medical Center with waiver of the requirement for obtaining informed consent (approval No. 443) and was registered at UMIN (ID; UMIN000037043). The study was conducted in accordance with the Declaration of Helsinki. Information from medical records was anonymized and deidentified prior to final analysis.

### Study population

We enrolled 255 patients with type 2 DM who visited the outpatient clinic of Tokyo Bay Urayasu Ichikawa Medical Center and had been treated with SGLT2 inhibitors between April 2014 and September 2018. Eligible subjects were aged 18 years or older and were either treatment-naïve (i.e., no glucose-lowering drugs) or on antidiabetic therapy before administration of SGLT2 inhibitors with HbA1c between 7.0% and 11.0%. Patients on steroid therapy or immunosuppressive agents were excluded from the study. Ultimately, 223 subjects were included in this study.

### Study design

The effects of SGLT2i on eGFR as well as systolic and diastolic BP were evaluated over 12 months. Because multiple antidiabetic and antihypertensive agents that could affect renal hemodynamics are commonly used as background medications, the effects of these drugs on SGLT2 inhibitor-induced changes in eGFR were also assessed. Biochemical parameters, including HbA1c, serum creatinine, LDL-cholesterol (LDL-C) and serum Mg, as well as anthropometric parameters (BP and BW), were assessed over 12 months following the administration of SGLT2 inhibitors. eGFR was calculated using the formula adapted to the Japanese population [10].

$${\mathrm{eGFR}} 	= {\mathrm{194}} \times {\mathrm{age}}^{{\mathrm{ - 0}}{\mathrm{.287}}} \\ 	\times {\mathrm{serum}}\,{\mathrm{creatinine}}^{{\mathrm{ - 1}}{\mathrm{.094}}}\left( { \times {\mathrm{0}}{\mathrm{.739,}}\,{\mathrm{if}}\,{\mathrm{female}}} \right)$$

### Statistical analysis

The results are expressed as the mean ± SE or median [lower quartile, upper quartile]. Data were compared by t-test or Mann-Whitney U test, as appropriate. Serial changes in eGFR over 12 months were assessed with a piecewise linear regression model in two time periods: 0 to 3 months and 3 to 12 months. Stepwise multiple regression analyses were applied to evaluate which parameters (age, sex, antidiabetic/antihypertensive drugs used, changes in HbA1c, systolic BP and BW) had a greater impact on the early changes (0 to 3 months) in eGFR induced by SGLT2 inhibitors. Statistical analyses were performed using Statistical Package for Social Sciences (SPSS) version 25 (IBM, www.ibm.com). P values less than 0.05 were considered statistically significant.

## Results

### Effects of SGLT2i on renal hemodynamics

The baseline characteristics of SGLT2 inhibitor-treated subjects showed that the mean age was 58.1 ± 0.8 y/o, and HbA1c levels were 7.89 ± 0.08% (Table 1). The patients’ eGFR was 73.2 ± 1.6 mL/min/1.73 m^2^ (Table 2), and 73.5% of the subjects exhibited relatively preserved renal function (eGFR ≥ 60 mL/min/1.73 m^2^).

**Table 1:** Baseline characteristics of patients

		Drug therapies	*N* (%)
*N* (male/female)	223 (149/74)	Antidiabetics	
Age (y/o)	58.1 ± 0.8	DPP-4 inhibitors	164 (73.5%)
Body weight (kg)	77.9 ± 1.5	Metformin	126 (56.5%)
BMI (kg/m^2^)	28.4 ± 0.5	Sulfonylurea	16 (7.2%)
HbA1c (%)	7.89 ± 0.08	GLP-1 agonists	10 (4.5%)
LDL-cholesterol (mg/dL)	108.4 ± 6.4	Insulin	65 (29.1%)
		Antihypertensives	
eGFR (mL/min/1.73 m^2^)	*N* (%)	RAS inhibitors	114 (51.1%)
≥90	44 (19.7%)	ARB	69 (30.9%)
90 > ≥60	120 (53.8%)	ACE inhibitors	45 (20.2%)
60 > ≥45	38 (17.0%)	Ca channel blockers	87 (39.0%)
45 > ≥30	13 (5.8%)	β blockers	45 (20.2%)
30 >	8 (3.6%)	Diuretics	22 (9.9%)
		Loop	18 (8.1%)
		Thiazides	4 (1.8%)

**Table 2:** Temporal changes in blood and kidney parameters

*N* = 223	Baseline	3 months	6 months	12 months
Body weight (kg)	77.9 ± 1.5	75.3 ± 1.5^***^	75.7 ± 1.6^***^	76.3 ± 1.5^*^
BMI (kg/m^2^)	28.4 ± 0.5	27.6 ± 0.5^*^	27.7 ± 0.5^*^	27.8 ± 0.4^*^
Systolic BP (mmHg)	138.2 ± 1.2	135.7 ± 1.4^*^	134.8 ± 1.3^*^	133.3 ± 1.4^**^
Diastolic BP (mmHg)	78.9 ± 1.0	77.4 ± 1.1	76.8 ± 1.0	76.6 ± 1.0
HbA1c (%)	7.89 ± 0.08	7.36 ± 0.06^***^	7.36 ± 0.06^***^	7.32 ± 0.07^***^
LDL-cholesterol (mg/dL)	108.4 ± 6.4	99.9 ± 2.0	100.1 ± 2.1	99.8 ± 2.1
Serum Mg (mg/dL)	1.93 ± 0.02	2.07 ± 0.02^**^	2.05 ± 0.02^**^	2.01 ± 0.02^**^
eGFR (mL/min/1.73m^2^)	73.2 ± 1.6	69.5 ± 1.6^***^	69.7 ± 1.6^***^	70.2 ± 1.7^*^
UACR (mg/g)	67.6 (14.6–411.7)	69.8 (16.0–281.0)	53.8 (13.8–301.5)^*^	60.4 (13.6–276.2)

UACR, urine albumin/creatinine ratio

*p < 0.05, **p < 0.01, ***p < 0.005 vs. baseline

The following SGLT2 inhibitors were given: dapagliflozin (32.7%), ipragliflozin (28.3%), canagliflozin (14.8%), empagliflozin (11.2%), luseogliflozin (8.1%) and tofogliflozin (4.9%). Other glucose-lowering drugs used concomitantly with SGLT2 inhibitors are shown in Table 1. Metformin and sulfonylurea were avoided in patients with moderately impaired renal function (Supplementary Table). For BP-lowering agents, RAS inhibitors were given to 51.1% of the subjects (angiotensin receptor blockers (ARBs); 30.9%, angiotensin-converting enzyme inhibitors (ACEis); 20.2%) prior to the administration of SGLT2 inhibitors. Thirty-nine percent of the subjects received CCBs, of which amlodipine was used in more than half of the patients (amlodipine; 22.0%, nifedipine CR; 6.7%, cilnidipine; 4.5%, azelnidipine; 3.6%, benidipine; 2.2%). β blockers were given to 20.2% of the subjects at the beginning of the study.

Administration of SGLT2 inhibitors reduced HbA1c (−0.53%, *p* < 0.001), BW (−2.6 kg, *p* < 0.001) and systolic BP (−2.5 mmHg, *p* < 0.05) at 3 months, and these changes were sustained throughout the study period (Table 2). Serum Mg was elevated following treatment with SGLT2 inhibitors (*p* < 0.01).

SGLT2 inhibitors elicited a decrease in eGFR at 3 months (from 73.2 ± 1.6 to 69.5 ± 1.6 mL/min/1.73 m^2^ *p* < 0.005), and this effect was sustained until the end of the study (Table 2). The rate of the changes in eGFR from 3 to 12 months was 1.1 ± 1.1 mL/min/1.73 m^2^/year. The urinary albumin-to-creatinine ratio was nearly unchanged throughout the study period.

### Effects of various parameters on initial eGFR dips

To elucidate what factors had a significant impact on the initial eGFR reduction induced by SGLT2 inhibitors, the correlations between the early decrease in eGFR (0 to 3 months) and various biochemical and anthropometric parameters were assessed with the use of stepwise multiple regression analysis. Thus, male sex, baseline eGFR and metformin use constituted significant determinants of the early changes in eGFR (male: *β* = −8.541, *p* < 0.005; baseline eGFR: *β* = 0.189, *p* < 0.005; metformin use: *β* = 6.406, *p* < 0.05). CCB use was marginally associated with the initial dip in eGFR (*β* = −5.780, *p* = 0.07). In contrast, age or changes in systolic BP, BW and HbA1c were not correlated with early decreases in eGFR.

### Effects of antidiabetic drugs on SGLT2 inhibitor-induced changes in eGFR

**Fig. 1:**
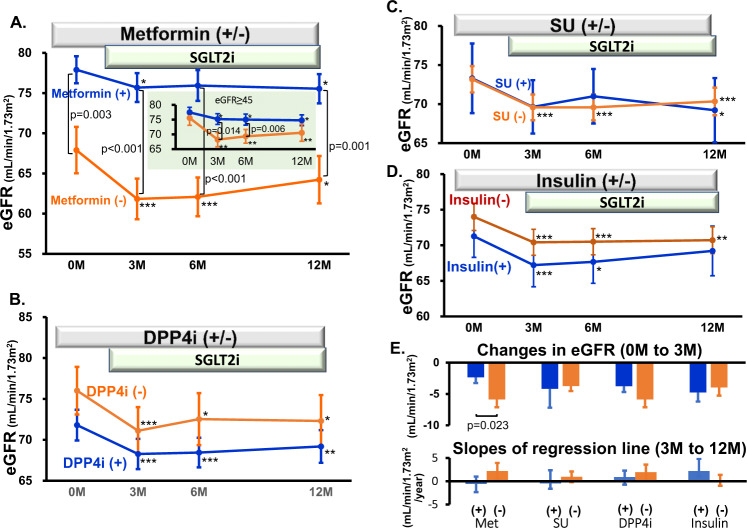
Effects of antidiabetic drugs on SGLT2 inhibitor-induced changes in eGFR. SGLT2i; SGLT2 inhibitors, DPP4i; DPP4 inhibitors, SU; sulfonylurea, Met; metformin. *p < 0.05, **p < 0.01, ***p < 0.005 vs. 0 month

Background use of metformin significantly prevented the SGLT2 inhibitor-induced decrease in eGFR (Fig. 1a, e). Thus, under no metformin treatment at baseline, SGLT2 inhibitors reduced eGFR by 5.9 ± 1.3 mL/min/1.73 m^2^ at 3 months (i.e., from 67.9 ± 2.9 to 61.8 ± 2.5 mL/min/1.73 m^2^, *p* < 0.005). In metformin users, however, SGLT2 inhibitor-induced changes in eGFR were blunted (77.9 ± 1.7 to 75.7 ± 1.8 mL/min/1.73 m^2^, *p* < 0.05), corresponding to approximately one-third of the response observed in patients who were not using metformin (*p* = 0.023). To eliminate the possibility that baseline renal function might affect eGFR responses to SGLT2 inhibitors, the effect of SGLT2 inhibitors on eGFR was assessed in patients with baseline eGFR ≥ 45 mL/min/1.73 m^2^; the baseline eGFR did not differ between metformin users and nonusers, and the blunted responses to SGLT2 inhibitors were again seen under metformin treatment. Changes in HbA1c, systolic BP or BW did not differ between these groups.

Other background medications, including DPP4i, sulfonylureas and insulin, had no effects on the changes in eGFR induced by SGLT2 inhibitors (Fig. 1b–e). None of the antidiabetic drugs used for background medications affected the long-term (3 to 12 months) changes in eGFR (Fig. 1e).

### Effects of antihypertensive drugs on SGLT2 inhibitor-induced changes in eGFR

#### RAS inhibitors

**Fig. 2:**
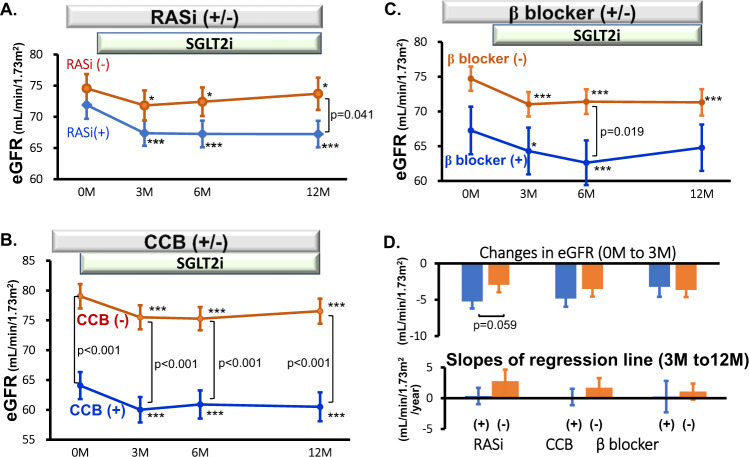
Effects of antihypertensive drugs on SGLT2 inhibitor-induced changes in eGFR. SGLT2i; SGLT2 inhibitors, RASi; renin-angiotensin system inhibitors, CCB; Ca^2+^ channel blockers. *p < 0.05, **p < 0.01, ***p < 0.005 vs. 0 month

**Fig. 3:**
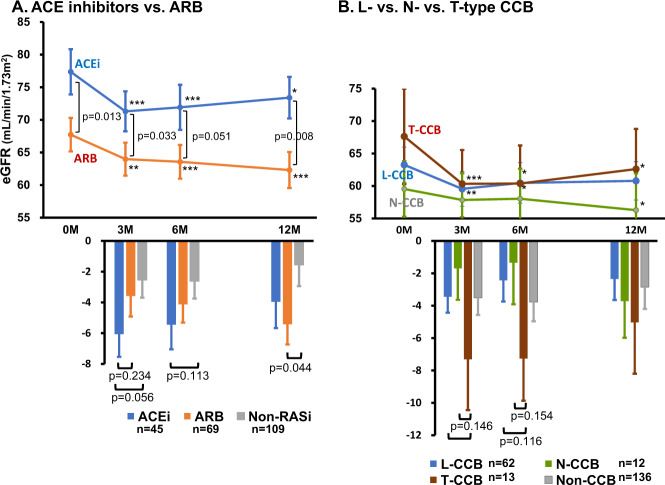
Effects of ACE inhibitors/ARB and Ca channel subtype blockers on SGLT2 inhibitor-induced changes in eGFR. ACEi; ACE inhibitors, ARB; angiotensin receptor blockers, CCB; Ca^2+^ channel blockers. Non-RASi; subjects not using ACEi or ARB. Non-CCB: subjects not using Ca^2+^ channel blockers. *p < 0.05, **p < 0.01, ***p < 0.005 vs. 0 month

Among 223 subjects, 114 patients had been given RAS inhibitors as background medications when SGLT2 inhibitors were started (Table 1). Baseline eGFR did not differ between the RAS inhibitor-treated and untreated groups (Fig. 2a). In subjects without RAS inhibitor treatment, the administration of SGLT2 inhibitors caused an initial decrease in eGFR from 74.6 ± 2.3 to 71.8 ± 2.4 mL/min/1.73 m^2^ at 3 months (*p* < 0.05), and the responses were sustained until the last visit. Under background treatment with RAS inhibitors, SGLT2 inhibitors also reduced eGFR, manifesting an initial decrease from 71.9 ± 2.2 to 67.4 ± 2.0 mL/min/1.73 m^2^ at 3 months (*p* < 0.005), which was marginally greater than that in patients not using RAS inhibitors (*p* = 0.059, Fig. 2d). When the effects of ACEis and ARBs on the SGLT2 inhibitor-induced changes in eGFR were compared, no difference was noted between these two subgroups, although the effect of ACEis tended to be greater than that of ARBs or non-RAS inhibitors at 3 months (Fig. 3a).

#### CCBs

Baseline eGFR was significantly lower in patients treated with CCBs than in those not given CCBs (*p* < 0.001, Fig. 2b). In patients using CCBs as background therapy at baseline, SGLT2 inhibitors substantially reduced eGFR from 64.1 ± 2.3 to 60.0 ± 2.1 mL/min/1.73 m^2^ at 3 months (*p* < 0.005). The SGLT2 inhibitor-induced changes in eGFR did not differ between the CCB-treated and untreated groups (Fig. 2d). Furthermore, to eliminate the confounding effect of different baseline eGFRs on the subsequent responses to SGLT2 inhibitors, the subjects were categorized into the groups with the following baseline eGFRs: ≥90, <90 and ≥60, <60 and ≥45, <45. Thus, no difference in the baseline eGFR or the response to SGLT2 inhibitors was seen between the CCB-treated and untreated groups for any of the eGFR categories (Supplementary Fig.).

CCBs are subclassified as L-, T- and N-type CCBs [11]. When the background effects of CCB subtypes on SGLT2 inhibitor-induced changes in eGFR were evaluated, the reduction in eGFR tended to be more pronounced under treatment with T-type CCBs than under L- or N-type CCB treatment (Fig. 3b).

#### β-blockers

In patients who had been treated with β blockers at baseline, the administration of SGLT2 inhibitors reduced the eGFR from 67.3 ± 3.4 to 64.3 ± 3.4 mL/min/1.73 m^2^ at 3 months (*p* < 0.05, Fig. 2c). The changes in eGFR by SGLT2 inhibitors did not differ from those in patients not using β blockers (−3.22 ± 1.37 vs. −4.17 ± 0.86 mL/min/1.73 m^2^, *p* > 0.5, Fig. 2d).

None of the background antihypertensive medications, including RAS inhibitors, CCBs and β blockers, modified the long-term (3 to 12 months) effects of SGLT2 inhibitors on eGFR (Fig. 2d).

### Interactions among antidiabetic and antihypertensive drugs

**Fig. 4:**
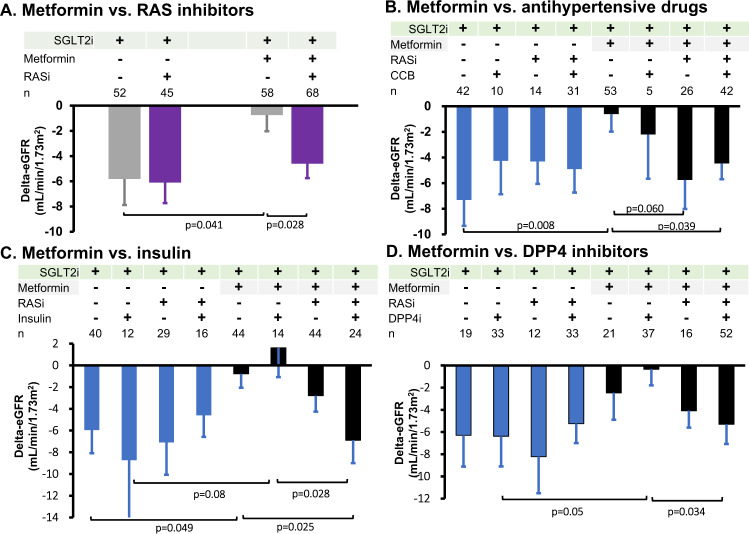
Interaction between SGLT2 inhibitors and antidiabetic/antihypertensive drugs on eGFR after 3 months of SGLT2 inhibitor therapy. SGLT2i; SGLT2 inhibitors, RASi; renin-angiotensin system inhibitors, CCB; Ca^2+^ channel blockers, DPP4i; DPP4 inhibitors

The interactive effects of antidiabetic and antihypertensive drugs on the changes in eGFR were assessed after 3 months of SGLT2 inhibitor treatment. In patients not taking metformin as a background medication, SGLT2 inhibitors decreased eGFR, which did not depend on concomitant use of RAS inhibitors (Fig. 4a). In contrast, under background treatment with metformin, the eGFR response to SGLT2 inhibitors was abolished in patients not using RAS inhibitors (from 79.9 ± 2.3 to 79.3 ± 2.7 mL/min/1.73 m^2^, *n* = 58, *p* > 0.5) but was preserved when RAS inhibitors had been given along with metformin for baseline therapy (from 75.9 ± 2.4 to 72.4 ± 2.4 mL/min/1.73 m^2^, *n* = 68, *p* < 0.01).

We further evaluated the effects of metformin, RAS inhibitors and CCBs on the eGFR responses to SGLT2 inhibitors (Fig. 4b). In patients not taking metformin, RAS inhibitors or CCBs as background medication at baseline, SGLT2 inhibitors caused a decrease of 7.3 ± 2.0 mL/min/1.73 m^2^ in eGFR (i.e., from 71.7 ± 4.8 to 63.9 ± 4.4 mL/min/1.73 m^2^, *n* = 42, *p* < 0.001). Likewise, the initial eGFR dip seen in patients using CCBs, RAS inhibitors or both but not metformin at baseline did not differ from that with SGLT2 inhibitors alone. In striking contrast, under background treatment with metformin but not CCBs or RAS inhibitors, SGLT2 inhibitors failed to reduce eGFR (from 81.0 ± 2.3 to 80.6 ± 2.8 mL/min/1.73 m^2^, *n* = 53, *p* > 0.5). Treatment with CCBs, along with metformin, did not cause significant improvement in the SGLT2 inhibitor-induced initial eGFR dip, but RAS inhibitors marginally recovered this response (*p* = 0.06). The combination of all three medications at baseline significantly restored the SGLT2 inhibitor-induced response of eGFR.

The interaction between SGLT2 inhibitors and other antidiabetic drugs (i.e., metformin, insulin and DPP4 inhibitors) was assessed after 3 months of SGLT2 inhibitor treatment. Again, among these antidiabetic drugs as background medications, only metformin, but not insulin or DPP4 inhibitors, affected the SGLT2 inhibitor-induced decrease in eGFR, which was restored by RAS inhibitors (Fig. 4c, d).

## Discussion

Abundant evidence has accrued indicating that SGLT2 inhibitors not only ameliorate glycemic control but also offer beneficial action on several vital organs, including the heart, liver and kidneys. Recent randomized trials, such as EMPA-REG OUTCOME [3], the CANVAS Program [4], DECLARE-TIMI58 [5] and CREDENCE [6], suggest that early reductions in eGFR following treatment with SGLT2 inhibitors are possibly mediated by enhanced TGF and are supposedly associated with improved renal outcomes in type 2 DM patients. In these trials, however, multiple antidiabetic and antihypertensive drugs were used as background medications, which could modify the renal hemodynamic action of SGLT2 inhibitors and might influence long-term renal outcomes. For example, in the EMPA-REG OUTCOME, CANVAS and CREDENCE trials, 80 to 100% of the subjects had received RAS inhibitors prior to the administration of SGLT2 inhibitors [3–6]. Likewise, metformin was used in 50 to 80% of the patients in combination with SGLT2 inhibitors. It appears relevant therefore to clarify the role of concomitant use of antidiabetic and antihypertensive drugs in the SGLT2 inhibitor-induced renal hemodynamic effects in patients with type 2 DM and hypertension.

The present study shows that SGLT2 inhibitors not only ameliorate glycemic control but also alter renal hemodynamics (Table 2). Furthermore, SGLT2 inhibitors reduce systemic BP and BW and increase serum Mg, which is consistent with the results demonstrated previously [3–6, 12]. In our current study, the patients received several classes of antidiabetic drugs, including DPP4 inhibitors (73.5%), metformin (56.5%), and insulin (29.1%), as background medications at baseline. We therefore evaluated the impact of these antidiabetic drugs on the eGFR responses to SGLT2 inhibitors. Thus, DPP4 inhibitors, sulfonylureas or insulin as background medication had no effect on the SGLT2 inhibitor-induced changes in eGFR (Fig. 1). In contrast, metformin markedly suppressed the renal hemodynamic response to SGLT2 inhibitors. Furthermore, to eliminate the possible confounding effects of RAS inhibitors and CCBs, the SGLT2 inhibitor-induced changes in eGFR were evaluated under the condition where no RAS inhibitor or CCB was given; metformin almost totally abolished the SGLT2 inhibitor-induced changes in eGFR (−0.61 ± 1.37 mL/min/1.73 m^2^, Fig. 4b). Several studies showed that the administration of metformin elevated eGFR [13, 14]. Furthermore, Rosenstock et al. [14] demonstrated that the SGLT2 inhibitor-induced reduction in eGFR was attenuated by the simultaneous administration of metformin, which agreed with our results and hence suggested the vasodilator property of metformin. In this regard, it is recognized that metformin stimulates AMP-activated protein kinase (AMPK) [15], which would facilitate nitric oxide production [16, 17]. The background treatment with metformin may therefore blunt the action of SGLT2 inhibitor-induced TGF via nitric oxide [18] and could lead to the diminished response of eGFR to SGLT2 inhibitors. Of note, van Bommel et al. [19] recently demonstrated that the dapagliflozin-induced activation of TGF and the decrease in eGFR are attributed to efferent arteriolar vasodilation rather than afferent arteriolar constriction in metformin-treated patients with type 2 DM. Thus, the efferent arteriolar dilation induced by SGLT2 inhibitors could be undermined under the condition where this arteriole is already dilated by metformin.

Although it remains fully undetermined to what extent the renal hemodynamic action of SGLT2 inhibitors contributes to improved renal outcomes in type 2 DM [20], metformin interference with the eGFR response to SGLT2 inhibitors might affect the progressive nature of DM nephropathy. Metformin per se is reported to cause beneficial [21] or detrimental effects on renal function in patients with type 2 DM [13, 22]. It has been demonstrated, however, that in the EMPA-REG OUTCOME trial, empagliflozin reduces the risks of progressive nephropathy but induces a diminished reduction in this risk for patients using metformin compared with those not using metformin (32% vs. 53% risk reduction, interaction *P* = 0.01) [8]. Similar observations have been reported concerning the cardioprotective effects of empagliflozin (29% vs. 54% risk reduction of cardiovascular death, interaction *P* = 0.07) [8] and canagliflozin (12% vs. 36% risk reduction of cardiovascular death or hospitalization, interaction *P* = 0.03) [9]. In this regard, Packer [23, 24] suggested that the beneficial effects of SGLT2 inhibitors that depend on AMPK are attenuated in the setting where AMPK has already been activated by background treatment with metformin. This interactive effect is noteworthy because combination therapy with metformin and SGLT2 inhibitors is commonly provided to a large number of patients with type 2 DM [3, 4, 6]. Nevertheless, even under background therapy with metformin, SGLT2 inhibitors should still offer substantial renal and cardiovascular benefits. Furthermore, therapeutic strategies for treating patients with DM and hypertension actually include RAS inhibitors [25, 26], which could restore the metformin-induced changes in renal hemodynamic action (Fig. 4).

RAS inhibitors are widely used in patients with DM and hypertension and are regarded as a first-line antihypertensive drug for DM [25, 26]. This class of agents exerts favorable action on renal outcomes, which is mediated at least in part by the amelioration of glomerular hypertension ascribed to efferent arteriolar dilation [27]. Similarly, SGLT2 inhibitors have emerged as a promising antidiabetic drug that confers additional benefits to alleviate progressive kidney disease, whose renal action is purportedly attributed in part to afferent arteriolar vasoconstriction due to enhanced TGF [28–30]. Intriguingly, van Bommel et al. [19] recently showed efferent arteriolar vasodilation by dapagliflozin as a crucial mechanism for reduced glomerular pressure in metformin-treated patients with type 2 DM; two-thirds of the patients had been given RAS inhibitors, and dapagliflozin caused decreases in both renal vascular resistance and GFR. It is anticipated therefore that the concomitant use of SGLT2 inhibitors and RAS inhibitors could act in concert to modulate the efferent arteriolar tone, resulting in the improvement in glomerular hypertension. In this regard, the post hoc analysis of the EMPA-REG OUTCOME by Mayer et al. [31] showed that in patients taking RAS inhibitors as a background medication at baseline, empagliflozin elicited a greater initial eGFR change than in those not taking RAS inhibitors. The present study also shows that background treatment with RAS inhibitors marginally enhances the SGLT2 inhibitor-induced decrease in eGFR (Fig. 2). Furthermore, under background treatment with metformin, the SGLT2 inhibitor-induced decrease in eGFR was abolished, but this response was efficiently preserved when RAS inhibitors had been given along with metformin for baseline therapy (Fig. 4a). Of note, it is well known that adenosine constitutes a crucial mediator of TGF, eliciting not only preglomerular vasoconstriction but also postglomerular vasodilation [32–34], especially in the presence of RAS inhibitors [32]. Collectively, these observations suggest an intimate interplay between SGLT2 inhibitors and RAS inhibitors acting on glomerular microvessels, whereby the renal hemodynamic effect of SGLT2 inhibitors is augmented.

Of interest, the present study shows that ACEis tend to augment the SGLT2 inhibitor-induced decrease in eGFR to a greater extent than ARBs (Fig. 3). It is well known that ACEi stimulates bradykinin activity [35], and we previously demonstrated that an ACEi caused increased vasodilator action on efferent arterioles than an ARB in dog kidneys, the mechanism of which involved enhanced intrarenal bradykinin activity [36]. Nevertheless, the long-term effects of these RAS inhibitors on SGLT2 inhibitor-induced changes in renal function need to be established.

CCB is a potent antihypertensive agent and is accepted as an add-on therapy to RAS inhibitors in hypertensive patients with DM. In the EMPA-REG OUTCOME trial, 27.8% to 45.8% of the patients received CCBs [3]. Because CCBs potently cause afferent arteriolar vasodilation [11, 37], CCBs could counter the afferent arteriolar constriction induced by SGLT2 inhibitor-induced augmentation of TGF. The post hoc analysis for the EMPA-REG OUTCOME, however, shows that CCBs do not modify the empagliflozin-induced decrease in eGFR [31]. Similarly, our present study demonstrates that treatment with CCBs has no inhibitory effect on the SGLT2 inhibitor-induced decrease in eGFR, irrespective of background medication with RAS inhibitors or metformin (Figs. 2 and 4). These observations are somewhat surprising because CCBs should render afferent arterioles refractory to the constrictor action of TGF. Of note, TGF has been shown to affect not only afferent but also efferent arteriolar tone in experimental animals, whose effects are mediated by intrarenal adenosine generation and the subsequent stimulation of adenosine A1 and A2 receptors [32–34]. Adenosine constricts afferent arterioles through adenosine A1 receptor-mediated stimulation of phospholipase C/inositol trisphosphate (IP3) production [38]. To the extent that IP3 stimulates Ca release from the sarcoplasmic reticulum and opening of Ca^2+^-activated chloride channels, leading to depolarization and Ca influx through voltage-dependent Ca channels [38–40], CCBs should block this vasoconstrictor mechanism. In contrast, efferent arterioles are devoid of L-type voltage-dependent Ca channels, a target for CCBs [11], but manifest vasodilation by adenosine through adenosine A2 receptors [32–34]. Because SGLT2 inhibitors increase urinary adenosine excretion in patients with type 2 [19] as well as type 1 DM [30, 41], adenosine could be responsible for SGLT2 inhibitor-induced efferent arteriolar vasodilation. Indeed, a recent study clearly shows that the SGLT2 inhibitor-induced decrease in eGFR is caused by postglomerular vasodilation rather than preglomerular vasoconstriction in metformin-treated type 2 DM patients [19]. It is judiciously anticipated therefore that SGLT2 inhibitors have the ability to decrease eGFR under the influence of CCBs.

Several subclasses of CCBs have been documented depending on the activity of different Ca channel subtypes. Nifedipine and amlodipine preferentially block L-type Ca channels and inhibit renal preglomerular (e.g., afferent) arteriolar tone [11]. Furthermore, efonidipine, azelnidipine and benidipine affect both preglomerular and postglomerular (i.e., efferent) arteriolar tone through blockade of L-type and T-type Ca channels [11, 42–44]. Finally, cilnidipine inhibits N-type Ca channels in the nerve terminals innervating afferent and efferent arterioles and hence dilates both microvessels [11, 44]. In the present study, we found a modest tendency of T-type CCBs to enhance the SGLT2i-induced reduction in eGFR (Fig. 3). Because of the paucity of patients treated with T-type and N-type CCBs, however, the interaction between CCB subtypes and SGLT2 inhibitors needs to be more thoroughly elucidated.

The present study also evaluated the influence of β blockers on eGFR responses to SGLT2 inhibitors. The results show that β blockers have no effect on the SGLT2 inhibitor-induced decrease in eGFR (Fig. 2). Furthermore, this effect was again blunted by background therapy with metformin (from 74.6 ± 3.9 to 72.7 ± 3.7 mL/min/1.73 m^2^, *n* = 22, *p* = 0.375) versus the absence of metformin (from 60.6 ± 5.2 to 56.3 ± 5.1 mL/min/1.73 m^2^, *n* = 23, *p* = 0.013). Other parameters, including HbA1c and BP, exhibited similar changes in response to SGLT2 inhibitors in the β blocker-treated and untreated groups.

Finally, the present study attempted to evaluate the effect of SGLT2 inhibitors on albuminuria and its association with changes in eGFR. Because of the nature of this study protocol (e.g., retrospective study), numerous missing data on albuminuria preclude its precise evaluation. Furthermore, since SGLT2 inhibitors upregulate sirtuin-1 (SIRT1) [45] and then mitigate glomerular injury through nonhemodynamic mechanisms, including the suppression of claudin-1 [46] and restored function of autophagy [47], hemodynamic parameters (i.e., eGFR and glomerular hypertension) may not necessarily parallel the renal pathophysiological process in DM nephropathy. Additionally, although the slopes of long-term (3 to 12 months) changes in eGFR do not differ between users and nonusers of specific background drugs, the relatively short observation period of this study (i.e., 12 months) abates the accuracy in assessing the temporal changes in renal function. More extensive studies would clarify the effect of SGLT2 inhibitors on albuminuria and long-term renal outcomes and could more precisely unveil the interaction of SGLT2 inhibitors with other antidiabetic and antihypertensive drugs.

In conclusion, the present study shows that background treatment with antidiabetic and antihypertensive drugs modifies the renal hemodynamic effects of SGLT2 inhibitors in patients with type 2 DM. Whereas the concomitant use of metformin and SGLT2 inhibitors confers greater benefits on glycemic control, there is a possibility that preceding treatment with metformin may diminish the SGLT2 inhibitor-induced decrease in eGFR. Further addition of RAS inhibitors to background medications, by contrast, restores this response. Finally, CCBs, though potent afferent arteriolar vasodilators, have no impact on the SGLT2 inhibitor-induced change in eGFR. Whether the renal hemodynamic effects of these background medications are linked to long-term renal outcomes requires further investigation.

## Supplementary information

Supplementary Figure

Supplementary Table
