# Supplementary material for: Effects of SGLT2 inhibitors on eGFR in type 2 diabetic patients—the role of antidiabetic and antihypertensive medications
Source: Hypertens Res. 2020 Dec 14;44(5):508–17. doi: 10.1038/s41440-020-00590-1 (PMC8099726; doi:10.1038/s41440-020-00590-1)
Supplement: Supplementary file 1 — Supplementary Figure [file 41440_2020_590_MOESM1_ESM.pptx]

## Slide 1
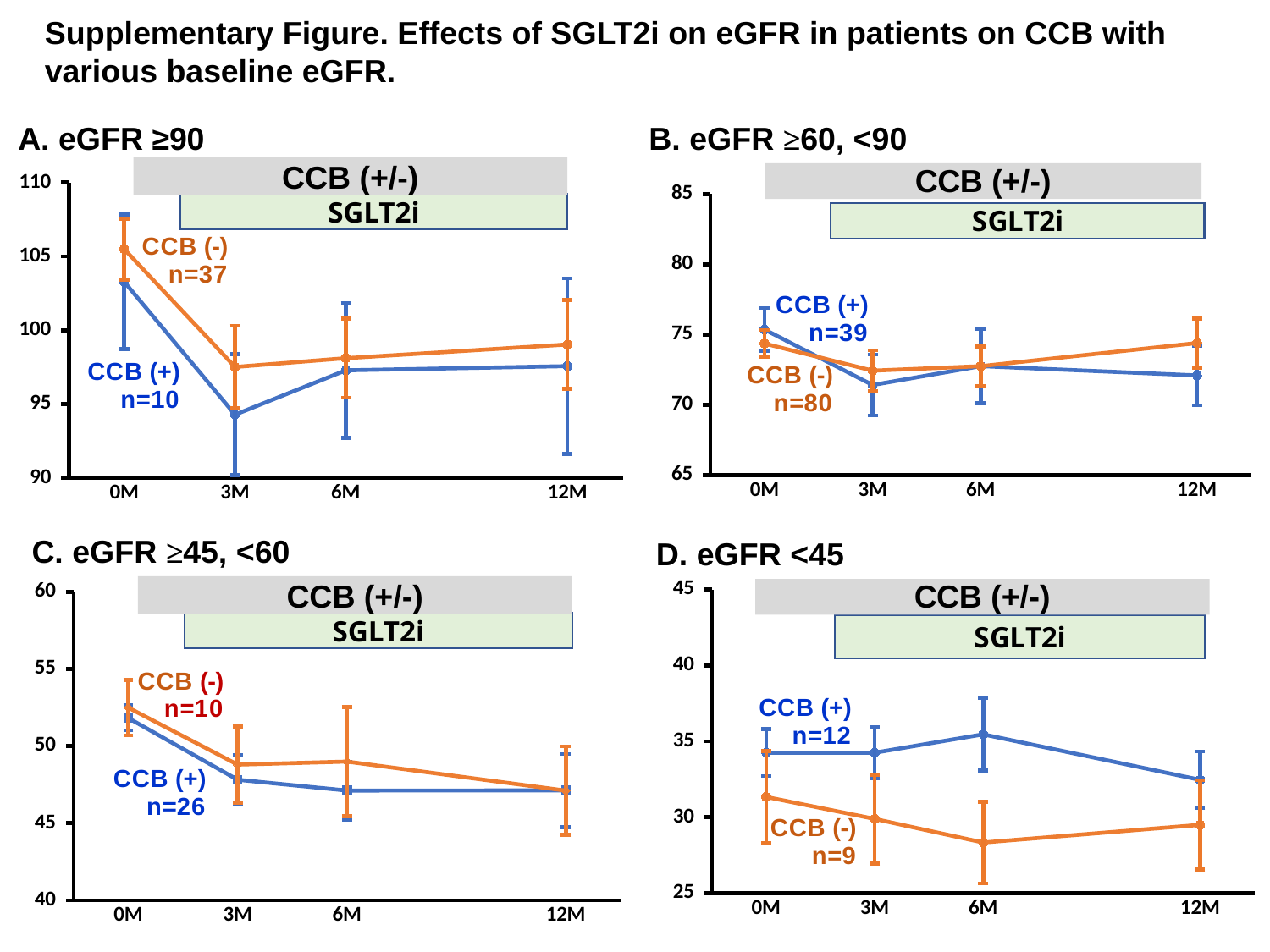

Supplementary Figure. Effects of SGLT2i on eGFR in patients on CCB with various baseline eGFR.
A. eGFR ≥90
B. eGFR ≥60, <90
### Chart
| Category | | |
|---|---|---|
| 0M | 75.36492902203318 | 74.36612071596227 |
| 3M | 71.4121205821088 | 72.4342810318562 |
| 6M | 72.7578947368421 | 72.75342465753425 |
| | None | None |
| 12M | 72.09729729729729 | 74.4 |CCB (+/-)
### Chart
| Category | | |
|---|---|---|
| 0M | 103.28571428571429 | 105.4904468622315 |
| 3M | 94.28571428571429 | 97.5154755405967 |
| 6M | 97.28571428571429 | 98.10810810810811 |
| | None | None |
| 12M | 97.57142857142857 | 99.03888888888889 |SGLT2i
C. eGFR ≥45, <60
D. eGFR <45
### Chart
| Category | | |
|---|---|---|
| 0M | 51.82647909567489 | 52.5 |
| 3M | 47.81669422503751 | 48.8 |
| 6M | 47.11538461538461 | 49.0 |
| | None | None |
| 12M | 47.125 | 47.111111111111114 |CCB (+/-)
### Chart
| Category | | |
|---|---|---|
| 0M | 34.253846153846155 | 31.333333333333332 |
| 3M | 34.25 | 29.88888888888889 |
| 6M | 35.46153846153846 | 28.333333333333332 |
| | None | None |
| 12M | 32.46153846153846 | 29.5 |SGLT2i
