# Supplementary material for: Effects of SGLT2 inhibitors on eGFR in type 2 diabetic patients—the role of antidiabetic and antihypertensive medications
Source: Hypertens Res. 2020 Dec 14;44(5):508–17. doi: 10.1038/s41440-020-00590-1 (PMC8099726; doi:10.1038/s41440-020-00590-1)
Supplement: Supplementary file 2 — Supplementary Table [file 41440_2020_590_MOESM2_ESM.pptx]

## Slide 1
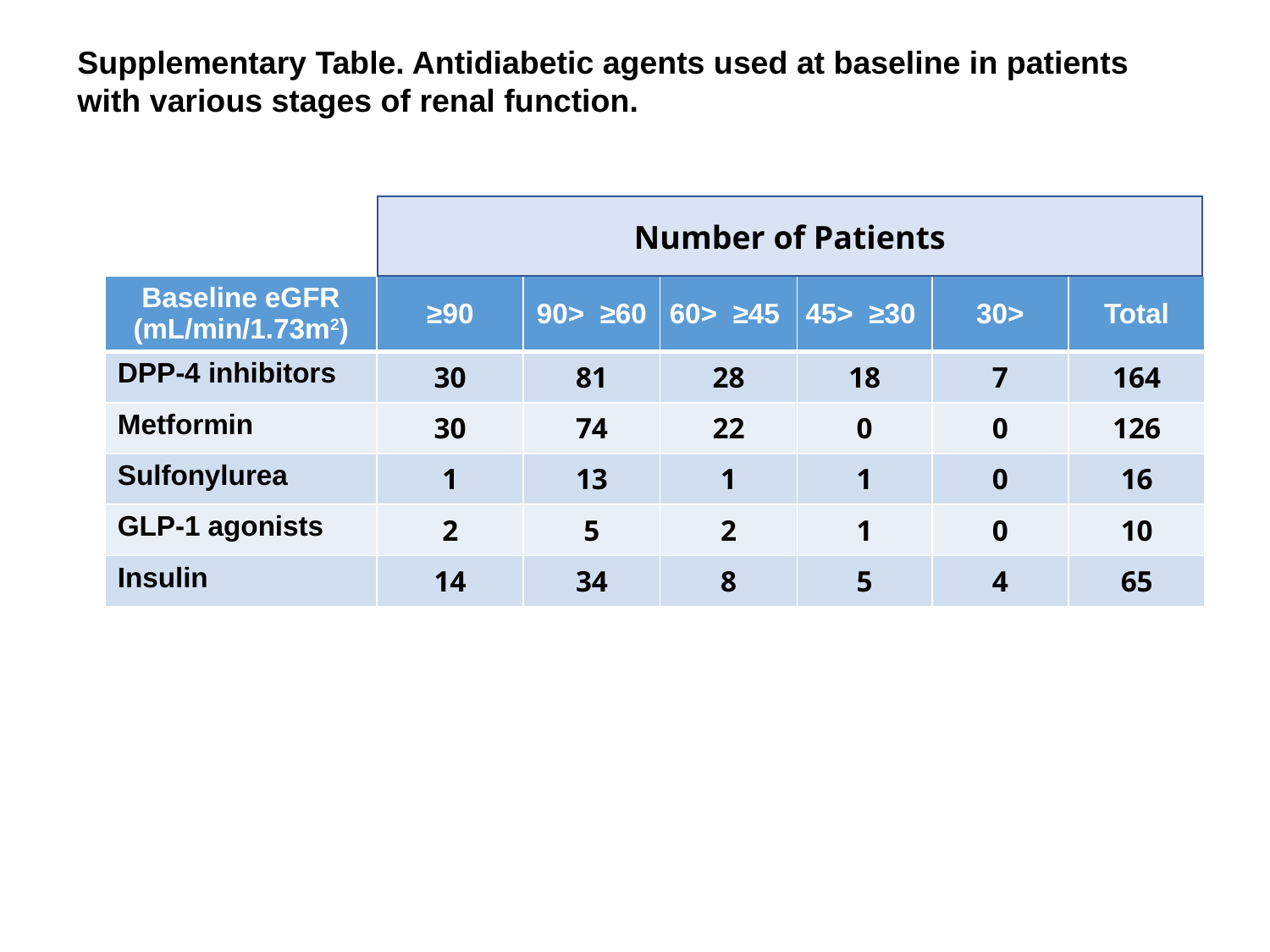

Supplementary Table. Antidiabetic agents used at baseline in patients with various stages of renal function.
Number of Patients
| Baseline eGFR (mL/min/1.73m2) | ≥90 | 90> ≥60 | 60> ≥45 | 45> ≥30 | 30> | Total |
| --- | --- | --- | --- | --- | --- | --- |
| DPP-4 inhibitors | 30 | 81 | 28 | 18 | 7 | 164 |
| Metformin | 30 | 74 | 22 | 0 | 0 | 126 |
| Sulfonylurea | 1 | 13 | 1 | 1 | 0 | 16 |
| GLP-1 agonists | 2 | 5 | 2 | 1 | 0 | 10 |
| Insulin | 14 | 34 | 8 | 5 | 4 | 65 |
